# Current status of and progress in the treatment of malignant pleural effusion of lung cancer

**DOI:** 10.3389/fonc.2022.961440

**Published:** 2023-01-20

**Authors:** Yuhua Zhao, Limeng Yu, Lili Wang, Yingxi Wu, Haiyang Chen, Qiming Wang, Yufeng Wu

**Affiliations:** Department of Internal Medicine, The Affiliated Cancer Hospital of Zhengzhou University & Henan Cancer Hospital, Zhengzhou, China

**Keywords:** malignant pleural effusion, lung cancer, angiogenesis, IPT, ATMPs-MTX

## Abstract

Malignant pleural effusion (MPE) is a common complication in the late stage of malignant tumors. The appearance of MPE indicates that the primary tumor has spread to the pleura or progressed to an advanced stage. The survival time of the patients will be significantly shortened, with a median survival of only a few months. There are a variety of traditional treatments, and their advantages and disadvantages are relatively clear. There are still many problems that cannot be solved by traditional methods in clinical work. The most common one is intrapleural perfusion therapy with chemotherapy drugs, but it has a large side effect of chemotherapy. At present, with the development of medical technology, there are a variety of treatment methods, and many innovative, significant and valuable treatment methods have emerged, which also bring hope for the treatment of refractory and recurrent MPE patients. Several clinical trials had confirmed that drug-carrying microparticles has less adverse reactions and obvious curative effect. However, there is still a long way to go to completely control and cure MPE, and the organic combination of clinical work and scientific research results is needed to bring dawn to refractory MPE patients.

## 1 Introduction

With advances in medical technology and in-depth research on the pathogenesis of malignant pleural effusion (MPE), innovative drugs and treatment strategies have been developed. MPE treatment has made many achievements. However, recalcitrant or recurrent MPE currently does not have effective treatment options. Therefore, the treatment of MPE is still a difficult clinical problem, and the current status and progress of MPE treatment are reviewed as follows.

Lung cancer accounts for 18.0% of all cancer deaths according to the latest 2020 Global Cancer Data Report from the World Health Organization’s International Agency for Research on Cancer (IARC) (1). The data show that approximately 50% of malignant tumors can present with malignant pleural effusion (MPE). MPE is more common in lung cancer, breast cancer and lymphoma, with rates as high as 75%, and lung cancer has the highest rate (2). MPE is one of the common complications of advanced malignant tumors, with a median survival of only 3-12 months (3–5).

Pleural fluid cytology is the easiest way to diagnose MPE and the gold standard for diagnosis. However, its sensitivity is limited in cases with few cancer cells, and the rate of positive detection (40%~87%) is relatively low (6); the rate of positive detection can be improved by testing pleural fluid samples multiple times or directly using pleural biopsy to detect cancer cells. For patients with clear primary tumors and asymptomatic MPE, no therapeutic intervention can be performed for the effusion itself (7–10). Once the amount of pleural effusion increases or if substantial pleural effusion is generated within a short period of time, it will cause symptoms such as cough, chest tightness, dyspnea and weakness, which will seriously affect the quality of life of patients. For MPE patients with obvious clinical symptoms, the primary aim of treatment is to relieve dyspnea, chest tightness and other symptoms (11, 12). The presence of MPE indicates that the primary tumor has spread to the pleura or has progressed to an advanced stage, and the survival of patients is significantly shortened. Once MPE is diagnosed in patients with tumors, it should be actively treated; otherwise, the accumulation of fluid will cause pulmonary atelectasis or recurrent lung infections and even threaten the life of the patient (13). With advances in medical technology and in-depth research on the pathogenesis of MPE, innovative drugs and treatment strategies have been developed. For example, the microparticles released by autologous tumor cells can be used to encapsulate chemotherapeutic drugs to achieve antitumor effects through two mechanisms: direct killing of tumor cells and activation of the autoimmune system (14, 15). However, recalcitrant or recurrent MPE does not currently have effective treatment options. Therefore, the treatment of MPE is still a difficult clinical problem, and the current status and progress of MPE treatment are reviewed as follows.

## 2 Routine clinical treatment modalities

Conventional clinical treatment mainly includes simple chest drainage, pleural fixation, thoracic thermal perfusion and intrathoracic drug infusion. Different treatment options have different indications and contraindications and different advantages and disadvantages, and clinicians usually conduct a comprehensive assessment to choose the most suitable treatment option for each patient.

### 2.1 Simple thoracentesis for fluid aspiration and tube placement for drainage

In patients with malignant tumors complicated with MPE, the tumor cells have spread or metastasized to the pleura at an advanced stage, and these patients have missed the opportunity for surgical treatment; in this case, internal palliative treatment is most commonly adopted in the clinic (16, 17). The primary task is to relieve respiratory distress and pain, and many clinicians will prioritize simple thoracentesis and aspiration or tube drainage. Thoracentesis alone can quickly relieve the symptoms of dyspnea, but it is a temporary treatment measure. The reason is that the intrathoracic pressure decreases significantly within a short period of time after fluid extraction, which in turn leads to fluid reunion, a faster increase in accumulated pleural fluid, and a higher recurrence rate (18). Repeated drainage of a large amount of pleural fluid can lead to hypoproteinemia, anemia, weakness, electrolyte disorders and other systemic symptoms (19, 20). In severe cases, circulatory collapse and death may occur. Therefore, simple chest puncture and drainage cannot solve the problem of recurrent massive pleural fluid in MPE and can actually accelerate the deterioration induced by the disease and lead to failure of primary tumor systemic treatment or poor efficacy.

### 2.2 Pleural fixation

Intrathoracic infusion of sclerosing agents, also known as pleural fixation, involves the use of sclerosing agents to chemically irritate and cause pleuritis, which causes adhesional atresia of the visceral and mural pleura and eventually leads to loss of the pleural space, causing a reduction in pleural fluid. The American Thoracic Society (ATS), in its latest edition of its MPE treatment guidelines issued in 2018 (7), recommends placement drainage or chemical pleural fixation as the preferred treatment option to relieve dyspnea in patients with symptomatic MPE without combined pulmonary atelectasis who have never been treated for MPE. The previous guidelines only recommended drainage as an option for patients with MPE combined with pulmonary atelectasis (10). The application of sclerosing agents under video-assisted thoracic surgery (VATS) is a common clinical method. And VATS talc poudrage is recommended for pleurodesis in patients with good performance status. The most commonly used drug in clinical pleural fixation is talcum powder (21–23). A randomized controlled trial has robustly demonstrated that there is no additional clinical effectiveness or cost-effectiveness benefit between talcum powder by thoracoscopy and talc slurry intercostal drainage for MPE patients (24). Therefore, talcum slurry and talcum powder have no difference in efficacy. It has the advantages of low cost and a high success rate compared with other sclerosing agents. The common side effects of talcum powder pleural fixation are fever and chest pain, which can be relieved in most patients but can cause serious adverse reactions in some patients, such as pulmonary edema, acute respiratory distress syndrome (ARDS), and acute respiratory failure (8, 25). In some cases, death can occur. Therefore, the application of talcum powder for pleural fixation for MPE has certain risks.

### 2.3 Thoracic thermal perfusion therapy

Thoracic thermal perfusion therapy takes advantage of the different tolerances of tumor cells and normal cells to different temperatures (26). Therefore, the key to successful thoracic thermal perfusion is to control the intrathoracic temperature, and conversely, if the temperature is not well controlled, the normal cells of the body will suffer much irreversible damage. Clinically, the intrathoracic temperature is usually maintained at approximately 43°C, which can damage and kill tumor cells without much interference with and impact normal cell function (27). In addition, the increase in intrathoracic temperature caused by thoracic heat perfusion can significantly dilate blood vessels, promote the absorption of chemotherapeutic drugs, significantly increase the concentration of drugs in the thoracic cavity, and increase the ability of drugs to kill tumor cells. Therefore, compared with intratoracic perfusion treatment with chemotherapeutic drugs alone, intratoracic thermal perfusion combined with chemotherapeutic drugs shows more advantages (28–30). On the one hand, chemotherapeutic drugs can directly kill tumor cells, resulting in a reduction in pleural fluid production, and on the other hand, the increase in temperature can expand the pleural blood vessels and promote the absorption of chemotherapeutic drugs by tumor cells, which greatly improves the drug utilization rate and chemotherapeutic drug efficacy. Within 24 h after the end of perfusion, almost all patients showed profuse sweating, hot flashes, elevated body temperature and increased heart rate, which were relieved by symptomatic treatment. However, thoracic thermal perfusion chemotherapy is generally not recommended for patients with a very poor systemic condition or those who are unsuitable for thoracic thermal perfusion, such as patients with obvious liver and kidney failure, severe cardiovascular or cerebrovascular diseases, poor healing of anastomosis after surgery, and extensive adhesions in the thoracic cavity.

### 2.4 Intrapleural perfusion therapy

The most rapidly advancing and preferred treatment option is intrapleural perfusion therapy (IPT), which is the most widely used strategy in clinical practice due to its obvious efficacy, simplicity and lack of serious adverse effects and is suitable for most patients with MPE (31). It can prolong the survival time and improve the quality of life for most MPE patients. Currently, there are many kinds of drugs used for IPT treatment, including chemotherapeutic drugs, immunomodulators, and Chinese patent medicines. Many innovative drugs are in clinical trials or in the development stage, giving new hope to patients with recalcitrant or recurrent MPE.

#### 2.4.1 Chemotherapy drugs

The chemotherapeutic drugs commonly used in clinical practice alongside thoracic infusion mainly include cisplatin, carboplatin, and bleomycin. Cisplatin, as a first-generation platinum drug, has strong antitumor activity and is thus more widely used in thoracic perfusion (32). Cisplatin can not only directly induce a local antitumor effect but also stimulate the pleura to cause pleurisy and pleural adhesions and cause chest occlusion; in addition, cisplatin can also be absorbed into blood circulation through the blood vessels on the pleura, which can inhibit primary foci and metastases and reduce pleural fluid in many ways (33). Moreover, this mode of drug delivery greatly improves the concentration of drugs in the chest cavity, reduces the toxic side effects caused by systemic chemotherapy, and is tolerated by most patients with mild adverse effects, making it the preferred treatment for MPE. Compared with the second-generation platinum drug carboplatin, the adverse effects of cisplatin mainly include gastrointestinal reactions and nephrotoxicity, with less bone marrow suppression. Typically, only a single chemotherapeutic agent is used clinically, but some investigators have combined multiple chemotherapeutic agents to enhance treatment efficacy by taking advantage of the synergistic effect of various drugs (34, 35). However, the toxic side effects of chemotherapeutic drugs, especially for many patients with advanced tumors who cannot tolerate them, for those who develop drug resistance after multiple doses, or for those with recalcitrant or recurrent MPE with poor response, can lead to the failure of local MPE treatment. Overall, chemotherapeutic agents administered *via* transthoracic infusion are effective, but resistance may occur after multiple doses, and there is limited overall efficacy and a high rate of pleural fluid recurrence.

#### 2.4.2 Biological response modifiers

The main mechanism of action of biological response modifiers is to stimulate inflammation in the plasma membrane, causing fibrosis of mesothelial cells and adhesions to occlude the pleural space, leading to a decrease in pleural fluid production. The most commonly used clinical biological response modifier is the Nocardia rubra cell wall skeleton (N-CWS). On the one hand, it can inhibit tumor cells and enhance the activity of macrophages, T cells and natural killer (NK) cells (36, 37). On the other hand, it can induce interferon, lymphokine-activated killer cell and tumor necrosis factor production and anticancer effects. Therefore, N-CWS has good clinical efficacy in patients with lung cancer with MPE, can significantly improve the immune function and survival rate of patients, and has mild toxic side effects, so it is worthy of wide clinical application. However, it should be used with caution in patients with MPE who already have high fever and allergic reactions, as it may aggravate existing symptoms and cause deterioration of the patient’s systemic condition.

## 3 Latest treatment advances

With the continuous development of tumor treatments and advances in antitumor drugs, the MPE treatment paradigm is being constantly modified, and an increasing number of new drugs and technologies are being applied in the clinic, such as antiangiogenic drugs, drug-carrying microparticles, and pleural bladder pumps. A large number of preliminary clinical studies have shown extraordinary efficacy and the ability to overcome some of the shortcomings of traditional treatment modalities and greatly reduce the toxic side effects caused by treatment, bringing new treatment strategies and modalities for MPE, especially for patients with MPE in whom existing treatments have been ineffective, for those with relapsed MPE, and for those who are resistant to traditional treatment methods. For patients with MPE who have relapsed or failed various treatments, indwelling pleural catheters are now clinically available.

### 3.1 Anti-angiogenic drugs

The generation, invasion and metastasis of malignant tumors and tumor angiogenesis are closely related (38). Therefore, inhibition of tumor neovascularization has become a new strategy for tumor therapy. The main antiangiogenic drugs are bevacizumab and recombinant human vascular endothelial growth factor (VEGF) inhibitors, both of which can be administered by transthoracic perfusion. Both of these antiangiogenic drugs can be combined with platinum agents, and this combination is more effective than platinum agents or antiangiogenic drugs alone, producing greater increases in the inhibition of tumor cells and better reducing the formation of effusion.

#### 3.1.1 Bevacizumab

VEGF is an important proangiogenic mediator, and VEGF/VEGFR-2 is an important signaling pathway for angiogenesis (39–44). The VEGF/VEGFR-2 axis mediates vascular endothelial cell proliferation and neovascularization (45), which leads to the production of pleural fluid (46). Bevacizumab, a human recombinant monoclonal antibody that mediates VEGF signaling, inhibits tumor angiogenesis, growth, and metastasis, reducing the generation and growth of blood vessels in the pleura and ultimately leading to a decrease in pleural fluid production. Tao et al. (47) retrospectively studied 21 patients with lung adenocarcinoma with MPE treated with bevacizumab combined with chemotherapy by intravenous infusion, and the MPE remission rate (RR) was 81.0%. The disease control rate (DCR) at 24 weeks was 89.5%, and 90.5% of patients experienced lung re-expansion after treatment. These results suggest that bevacizumab in combination with chemotherapy has significant efficacy and safety advantages for treating MPE in lung adenocarcinoma and is an option for patients with lung adenocarcinoma with MPE. The results of a study of patients with nonsquamous non-small-cell lung cancer with poorly controlled MPE after drainage tube placement or pleural fixation receiving bevacizumab in combination with chemotherapy showed a pleural effusion control rate (PECR, defined as the percentage of patients with no reaccumulation of MPE at 8 weeks) of 80%, pleural progression-free survival (PPFS) of 16.6 months, and overall survival (OS) of 19.6 months, and patients’ quality of life significantly improved (48).

Many clinical studies (49–52) have also tried to explore the efficacy and safety of intrathoracic injection of bevacizumab combined with platinum-based drugs in the treatment of MPE, and the results of the studies have shown that intrathoracic injection of bevacizumab combined with platinum-based chemotherapeutic drugs showed increased overall efficacy (the difference is statistically significant, P<0.05) compared with administration of platinum-based drugs alone; the RR of the bevacizumab combined with cisplatin group can be as high as 83.33%, significantly higher than the 50.00% of the cisplatin group. In addition, intrapleural injection of bevacizumab reduced the level of VEGF in pleural fluid, with milder, tolerable toxic effects. Single-agent anti-vascular therapy is not ideal (53), and the combination of bevacizumab with platinum drugs in the treatment of MPE significantly increases the therapeutic effect compared with monotherapy. A meta-analysis (54) pooled data from 71 patients with non-small-cell lung cancer with MPE and, for the first time, evaluated the therapeutic effect of intrathoracic injection of different doses of bevacizumab in patients with non-small-cell lung cancer with MPE. The efficacy of low-dose bevacizumab was not inferior to that of high-dose bevacizumab, and the use of low-dose bevacizumab significantly reduced the incidence of adverse events and toxic side effects, suggesting that intrathoracic injection of low-dose bevacizumab can be a suitable treatment for patients with non-small-cell lung cancer with MPE. Currently, there are no uniform standards for the administration, dosing and duration of bevacizumab treatment in patients with MPE, and there are many controversies regarding which specific regimen should be used for the treatment of MPE. Some investigators (55) believe that bevacizumab is effective whether administered intravenously or by thoracic infusion, but the evidence for the use of bevacizumab in the treatment of MPE remains flawed due to study design biases and the small number of subjects.

#### 3.1.2 Recombinant human VEGF inhibitors

Researchers have found that recombinant human VEGF inhibitors can downregulate the expression of VEGF and receptors (56), block VEGF and VEGFR tyrosine phosphorylation, and induce MMP expression (57). A recombinant human VEGF inhibitor was found to inhibit the production of blood vessels and lymphatic vessels in animal models (58). In 2015, Wei et al. (59) found that recombinant human VEGF inhibitors could only inhibit the production of effusion but not cause apoptosis or inhibit tumor growth. However, in recent years, investigators (60, 61) have concluded that recombinant human VEGF inhibitors can also inhibit tumor cell proliferation and induce tumor cell apoptosis. The combination of recombinant human VEGF inhibitors with platinum-based drugs exerts a synergistic effect, and combined administration is better than administration of platinum-based drugs alone (34). Combined administration can improve the quality of life of patients. On the one hand, platinum drugs can directly act on tumor cells and interfere with tumor cell DNA replication and transcription, thus inducing tumor cell necrosis. On the other hand, recombinant human VEGF inhibitors can promote the immune response, improve the local tumor microenvironment (62, 63), promote normalization of tumor vascular function (64), more effectively promote the delivery of platinum drugs to the tumor tissue (65, 66), and more effectively kill tumor cells. In a study evaluating the clinical efficacy and safety of a recombinant human VEGF inhibitor combined with chemotherapy for MPE in lung adenocarcinoma, the treatment group was given chemotherapy and recombinant human VEGF inhibitor *via* intrathecal administration, and the control group patients were given the same chemotherapy as the treatment group. The efficacy rates were 81.82% and 64.52% in the treatment and control groups, respectively (statistically significant difference, P=0.027). The MPE control rates (DCRs) were 93.94% and 79.03%, respectively (statistically significant difference, P=0.013). Dyspnea symptoms were significantly improved in the treatment group, and side effects were not significantly different between the two groups (67).

### 3.2 Drug-carrying microparticles

Normally, cellular microparticles in the human body are used to store, transport and digest cellular products and wastes and are important carriers for the transport of various substances (68). Researchers have used autologous tumor cell-derived microparticles (ATMPs) as novel individualized drug carriers (69–73). In other studies, ATMPs have been used as novel individualized drug carriers to deliver chemotherapeutic drugs to tumor cells in a targeted manner (74–76). These drugs can not only directly interfere with the proliferation of tumor cells but also activate antitumor immunity (14, 77–79). In addition, ATMPs can be used to overcome the killing of normal cells by chemotherapeutic drugs and the resistance of tumor cells to chemotherapeutic drugs. The mechanism by which ATMPs encapsulating methotrexate (ATMPs-MTX) activate the neutrophil response as a treatment for MPE has been studied (80). ATMPs-MTX trigger neutrophil recruitment through activation of CXCL1 and CXCL2 released from macrophages (15, 81, 82). ATMPs also reverse drug resistance in cancer stem cells (CSCs). CSCs take up ATMPs-MTX better than do differentiated cancer cells, leading to CSC death (83). Guo et al. (84) demonstrated through mouse models and human experiments that ATMPs encapsulating chemotherapeutic drugs have almost no toxicity and have largely reduced toxic side effects compared with chemotherapeutic drugs in clinical applications. The ORR of 11 patients with advanced lung cancer with MPE treated with ATMPs-MTX was 90.91%, and the median survival time (MST) was 240 days, demonstrating excellent efficacy and only minor side effects. The low level of toxic side effects induced by ATMPs encapsulating chemotherapeutic drugs makes them a promising option for MPE treatment. Currently, many hospitals have used drug-carrying microparticles for MPE treatment. Although their efficacy and safety have been clinically validated, more subjects and clinical studies are needed to further evaluate their efficacy. There are still some concerns regarding the use of drug-carrying microparticles in oncology and MPE treatment. Primarily, the safety of microparticles needs to be determined; for example, ATMPs may contain oncogenic factors that may contribute to tumor progression (85).

### 3.3 Pleural bladder pump

Astoul et al. (86) performed an in-depth study of a peritoneal bladder pump for the treatment of ascites. The scholars first proposed and designed the pleural bladder pump for the treatment of MPE and named it the pleurapump system, whose specific mechanism is to drive the transfer of accumulated fluid from the pleural cavity to the bladder, from where it can drain from the body *via* the urinary system. The pump has pressure and position sensors on it to regulate the flow rate and drainage of the effusion and to monitor and record the amount of pleural fluid drained, which is very useful. Previous research on the peritoneal bladder pump (Alfapump system) has yielded some results (87, 88). These successes have inspired researchers to study pleural effusion. However, Astoul et al. conducted only 2 clinical trials, and both subjects experienced varying degrees of dyspnea after implantation of the pleural bladder pump. The investigators suspect that these outcomes may be related to pump dysfunction due to catheter obstruction (86). Studies of the pleurapump system for MPE treatment are still in the exploratory phase, and more subjects and clinical studies are needed to explore the efficacy and safety of this system in the future.

### 3.4 Indwelling pleural catheter

The currently recommended approaches for recurrent symptomatic MPE are indwelling pleural catheter (IPC) placement and pleural fixation, but IPC placement is significantly superior to repeat thoracentesis or tube placement for drainage and has been shown to be a powerful palliative treatment for patients with recurrent or treatment-resistant MPE. IPCs can be inserted and tunneled through the skin into the pleural cavity, allowing intermittent drainage and promoting pleural fixation. IPC placement is simple to perform and can usually be performed on an outpatient basis (89). IPCs are an effective means of controlling recurrent MPE, especially for patients with pulmonary atrophy and atelectasis who wish to have a shortened hospital stay (90). Thomas et al. (91) conducted a multicenter, randomized controlled clinical trial that included 144 patients with MPE. The researchers showed a reduction in the number of hospital days after IPC treatment compared with after talc pleural fixation, and there was no statistically significant difference in efficacy, in line with the findings of Davies, Ost et al. (92, 93) The results of the study were consistent. Data from a multicenter, randomized, open-label clinical trial suggest that IPCs are more effective in facilitating spontaneous pleural fixation and may improve quality of life (94). Significant improvement of dyspnea symptoms and fewer complications after IPC treatment were seen (95). Compared to talc pleural fixation, IPC placement has a very high safety profile (96): the incidence of pleural infection is <5% (patients usually respond to antibiotic therapy, and catheter removal is usually not necessary); prolonged, intermittent drainage of exudative pleural effusions or celiac disease may cause significant protein loss, leading to systemic malnutrition; and fibrin clots in the catheter lumen can lead to obstruction. In 2018, Bhatnagar et al. (97) studied the treatment of MPE by outpatient IPC placement combined with talcum powder and found that the odds of pleural fusion were significantly higher than those associated with IPC placement alone; in addition, serious adverse effects were rare and generally well tolerated by patients.

## 4 Summary and outlook

In summary, MPE is a common complication of advanced malignant tumors, and its appearance often indicates poor prognosis and short survival, which seriously affects patient quality of life. Furthermore, poorly controlled MPE can seriously affect the primary tumor systemic treatment plan, so MPE treatment is especially important in tumor treatment. At present, MPE is mainly treated medically, and intrathoracic infusion is the main strategy. With the continuous development of intrathoracic infusion drugs, many kinds of drugs are available, including chemotherapeutic drugs, immunomodulators, traditional Chinese medicines, antiangiogenic drugs and drug-carrying microparticles. MPE patients have benefited greatly from these novel therapies, which have shown good efficacy in clinical application, and clinical symptoms such as dyspnea and wheezing have been greatly improved. Currently, there are many means of MPE treatment, but there is no standard treatment protocol, especially for patients with recalcitrant or relapsed MPE, who suffer from limited overall treatment efficacy, which seriously affects the OS of patients. Therefore, although good results in the treatment of MPE have been achieved, especially *via* the use of highly beneficial antiangiogenic therapies and immunotherapies, the treatment of MPE, especially recalcitrant or relapsed MPE, is still a clinical challenge, and many issues remain to be solved in the future. Therefore, it is important to explore the pathogenesis of MPE and combine treatment modalities and new therapeutic approaches to improve the quality of life and prolong the survival of MPE patients.

## Author contributions

All authors listed have made a substantial, direct, and intellectual contribution to the work and approved it for publication.
